# Antimicrobial resistance, virulence profile, and genetic analysis of ESBL-producing *Escherichia coli* isolated from Nile tilapia in fresh markets and supermarkets in Thailand

**DOI:** 10.1371/journal.pone.0296857

**Published:** 2024-01-12

**Authors:** Woranich Hinthong, Varangkana Thaotumpitak, Jarukorn Sripradite, Nitaya Indrawattana, Thassanee Srisook, Thida Kongngoen, Edward R. Atwill, Saharuetai Jeamsripong

**Affiliations:** 1 Princess Srisavangavadhana College of Medicine, Chulabhorn Royal Academy, Bangkok, Thailand; 2 Research Unit in Microbial Food Safety and Antimicrobial Resistance, Department of Veterinary Public Health, Faculty of Veterinary Science, Chulalongkorn University, Bangkok, Thailand; 3 Department of Social and Applied Science, College of Industrial Technology, King Mongkut’s University of Technology North Bangkok, Bangkok, Thailand; 4 Department Microbiology and Immunology, Faculty of Tropical Medicine, Mahidol University, Bangkok, Thailand; 5 Department of Population Health and Reproduction, School of Veterinary Medicine, University of California, Davis, Davis, CA, United States of America; Jaramogi Oginga Odinga University of Science and Technology, KENYA

## Abstract

This study investigated the prevalence and antimicrobial resistance (AMR) of *Escherichia coli* (*E. coli*) in Nile tilapia from fresh markets and supermarkets. A total of samples (*n* = 828) were collected from Nile tilapia including fish flesh (*n* = 276), liver and kidney (*n* = 276), and intestine (*n* = 276). Overall prevalence of fecal coliforms (61.6%) and *E. coli* (53.0%) were observed. High prevalence of *E. coli* was found in the intestine (71.4%), followed by the liver and kidney (45.7%). The highest prevalence of resistance was commonly found against tetracycline (78.5%), ampicillin (72.8%), and sulfamethoxazole (45.6%) with resistance to only tetracycline (15.2%) as the most common antibiogram. The prevalence of multidrug resistance (MDR) (54.4%) and Extended-spectrum beta-lactamases (ESBLs) (5.7%) were examined. The predominant virulence genes (*n* = 158) were *st* (14.6%), followed by *eaeA* (0.6%). The *bla*_TEM_ (73.4%), *tetA* (65.2%), and *qnrS* (57.6%). There is statistical significance between Nile tilapia from fresh markets and supermarkets. Based on logistic regression analysis, ampicillin-resistant *E. coli* was statistically associated with the phenotypic resistance to tetracycline and trimethoprim, and the presence of *bla*_TEM_ and *tetA* (*p* < 0.05). Further investigation of AMR transference and their mechanisms is needed for AMR control.

## Introduction

Antimicrobial resistance (AMR) is a crisis to global public health due to direct and indirect associated high morbidity and mortality of infected cases. In 2019, it was reported that there were 2.8 millions of AMR cases per year in the U.S. and AMR can cost 20 billion USD of healthcare-associated treatment [1, 2]. Without any effective control strategies, AMR will cause 10,000,000 deaths by 2050 with an estimated cost relating to AMR infection treatment around 100 trillion USD [3]. The study in 2012 done in Thailand reported that AMR causes over 80,000 cases and 38,000 deaths per year [4]. High prevalence of AMR in common human pathogens including *Staphylococcus aureus*, *E. coli*, *Klebsiella pneumoniae*, *Acinetobacter baumannii*, and *Pseudomonas aeruginosa* has been reported with ranging from 27–52% in Thailand [5]. It is estimated that the cost of treatment of these bacteria is 0.5 billion USD per year [4].

Both pathogenic and non-pathogenic bacteria can exhibit AMR. Bacteria that showed resistance equal to or more than 3 classes of antibiotic is called multidrug-resistant (MDR) bacteria, while bacteria that resist to all except 2 classes of antibiotics is termed as extensively drug-resistant (XDR) bacteria, and bacteria that resist to all antibiotics available is termed pandrug-resistant (PDR) bacteria [6]. AMR bacteria usually harbor antibiotic resistant genes that coordinate with the group of antibiotics they resisted to. For example, bacteria that showed resistance to tetracycline normally harbor tetracycline resistance gene (*tet*), with 40 different genes have been currently characterized [7]. *E. coli* is a bacterium in *Enterobacteriaceae* group, which is one of the most important bacteria that can cause diarrheagenic disease in humans through fecal-oral transmission. Therefore, it has been used as the determinant of fecal contamination in food production. Pathogenic *E. coli* that frequently contaminate food in Thailand are Enteropathogenic *E. coli* (EPEC), Enterotoxigenic *E. coli* (ETEC), Enteroaggregative *E. coli*, and Enterohemorrhagic *E. coli* (EHEC) or Shiga-toxin producing *E. coli* (STEC) [4, 8]. These pathogenic *E. coli* have specific virulence factors that are vital component to cause pathogenesis in humans and animals. The intimin producing gene, *eaeA*, corresponds to EPEC, while heat labile toxin gene (*lt*) and heat-stable toxin gene (*st*) correspond with ETEC, and shiga toxin 1 (*stx1*) and shiga toxin 2 (*stx2*) encoding genes correspond with STEC [9]. *E. coli* can be an important reservoir of antibiotic resistance genes since they can exhibit more than one gene in the different plasmid types which can be transferred to neighbor bacteria through conjugation. Extended-spectrum β-lactamases (ESBLs) are a group of enzymes that bacteria produce and confer to resistance of most beta-lactam antibiotics, including monobactam, aztreonam, and cephalosporins. Moreover, ESBL-producing bacteria can exhibit co-resistance to multiple classes of antibiotics [10]. The emergence of ESBL-producing *Enterobacteriaceae*, especially non-typhoidal *Salmonella* spp. and *E. coli*, became one of the crucial public health concerns, and subsequently fewer treatment options [11]. ESBLs bacteria harbor specific resistance genes on plasmids [12]. The most common genes found in ESBL bacteria in human and animal origin include *bla*_TEM_, *bla*_CMY-2_, and *bla*_OXA_ [13, 14]. Infection with ESBL bacteria can be more severe and prolonged hospital stay. In some severe ESBL-infected cases, last-resort antimicrobials are necessary for treatment of infected cases [15].

Nile tilapia (*Oreochromis niloticus*) is commonly cultivated worldwide due to its rapid growth, adaptability, and good commercial price [16]. The production of Nile tilapia increases dramatically from 2,658 thousand tons in 2010 to 4,525 thousand tons in 2018 [17]. Nile tilapia is a non-native fish species in Thailand and was introduced in 1965 and distribute to farmers by the Department of Fisheries [18]. Therefore, most of the Nile tilapia sold in fresh markets and supermarkets in Thailand come from the fish farming in cages or ponds. In Thailand, Nile tilapia is one of the most important cultured fish in the country with a production of 337,500 metric tons [19]. Exporting tilapia products from Thailand has been ranked among the top 6 countries in Asia, with 0.2 million tons in 2018 [17]. To yield maximum tilapia production, an integrated fish farming system with high stock density of fish can lead to a wide range of bacterial diseases and subsequently contribute to heavy and prolonged use of antimicrobials in tilapia farms. This could contribute to promoting the selection of resistant bacteria and developing MDR bacteria [20].

Thailand’s National Strategic Plan (NAP) on AMR 2017–2021 was established in 2016, and one of the main goals is to reduce the use of antimicrobials in animals, including the aquaculture sector. The Department of Fisheries of Thailand carried out the action plans by initiating AMR national surveillance program in fishery products, building microbiology laboratory capacity, and promoting antibiotic stewardship to farmers, in combination with farm biosecurity and disease prevention [21]. Even though the distribution of AMR in aquaculture has been reported worldwide, available publications regarding AMR monitoring and surveillance in Nile tilapia remain largely unclear in Thailand. Therefore, this study aimed to explore the prevalence of fecal coliforms and *E. coli* in meat, kidney and liver, and intestine of Nile tilapia, to examine virulence genes and ESBL-producing *E. coli*, and phenotypic and genotype of AMR *E. coli*, and to identify the association between AMR, ESBLs, and virulence among *E. coli* isolates.

## Materials and methods

### Sample collection and preparation

Two hundred and seventy-six Nile tilapia (*O. niloticus*) were collected from October 2019 to November 2020. Average weight of Nile tilapia was between 244.0 and 1,323.3 g. The Nile tilapia were retrieved from fresh markets (*n* = 151) and supermarkets (*n* = 125) in seven districts of Bangkok. The samples were collected from Din Daeng (*n* = 60, 21.7%), Pom Prap Sattru Phai (*n* = 58, 21.0%), Khlong San (*n* = 49, 17.8%), Samphanthawong (*n* = 38, 13.8%), Bangkok Yai (*n* = 29, 10.5%), Bang Sue (*n* = 29, 10.5%) and Thonburi (*n* = 13, 4.7%) based on the density of human population. The tilapia samples were placed into a sterile plastic bag and stored at < 10°C during transportation. The samples were submitted to the laboratory for bacterial isolation and confirmation within 6 h after collection.

The scales of all Nile tilapia samples were aseptically removed at the dissected area and samples were divided into 25 g of fish meat (*n* = 276), one g of liver and kidney (*n* = 276), and one g of intestine (*n* = 276) to receive a total sample size of 828. All specimens were individually placed into a sterile plastic bag for further analyses. The animal study was reviewed and approved by the ethics committee of Chulalongkorn University Animal Care and Use Committee (IACUC; Approval No. 2031048).

### Determination of fecal coliforms and *E. coli*

The samples were examined for fecal coliforms and *E. coli* followed by the United States Food and Drug Administration’s Bacteriological Analytical Manual [22]. Approximately, 225 ml of Buffered Peptone Water (BPW) (Difco, MD, USA) was added to fish meat samples, and 9 ml of BPW was separately added to liver and kidney, and intestine samples. The samples were homogenized using Stomacher 400 Circular lab blender (Seward, TX, UK). One ml of the mixed BPW suspension was added into 9 ml of lactose broth (Difco) containing a Durham tube. The samples were incubated at 37°C for 24 h to observe gas production and turbidity. One loopful of positive lactose broth suspension was added to EC Broth (Difco) and incubated at 44.5°C overnight. Turbid tubes with gas production were reported as fecal coliforms positive. A loopful of EC suspension was streaked on Levine-Eosin-Methylene Blue (L-EMB) agar (Difco) and MacConkey agar (Difco) plates. All the plates were incubated overnight at 37°C. Suspected colonies of *E. coli* were flat and dark-centered colonies with or without metallic sheen on EMB agar and pink colonies on MacConkey agar. Suspected colonies on EMB and MacConkey agar were then biochemically confirmed using indole test and triple sugar iron agar (Difco) test.

### Antimicrobial susceptibility testing

All bacterial strains were analyzed for minimum inhibitory concentrations (MICs) by a 2-fold agar dilution technique according to the Clinical and Laboratory Standards Institute (CLSI) [23]. Eight antimicrobials were selected as representatives of different antibiotic classes based on their importance in human and veterinary medicine. Their breakpoints (range of tested concentrations) were as follows: ampicillin (32, 0.125 to 512 μg/ml), chloramphenicol (32, 0.125 to 256 μg/ml), ciprofloxacin (4, 0.00195 to 64 μg/ml), gentamicin (8, 0.25 to 128 μg/ml), streptomycin (32, 0.5 to 256 μg/ml), sulfamethoxazole (512, 0.5 to 2048 μg/ml), tetracycline (16, 0.0625 to 256 μg/ml), and trimethoprim (16, 0.25 to 256 μg/ml). MDR was classified as resistance to at least three groups of antimicrobials. *E. coli* ATCC 25922, *S. aureus* ATCC 25923, and *P. aeruginosa* ATCC 27853 reference strains were used as the quality control.

### ESBLs screening and confirmation

The detection of ESBL-producing *E. coli* isolates was performed using disk diffusion method [23]. This method divides into two phases, screening and confirmation test. In screening test, *E. coli* isolates were cultured in Muller-Hinton agar (MHA) (Difco) at 37°C overnight. Three antimicrobial disks of ceftazidime (30 μg), cefotaxime (30 μg), and cefpodoxime (10 μg) were placed on MHA agar plates and incubated at 37°C overnight. Bacterial isolates showing the resistance to at least one antibiotic were further confirmed using combination disk diffusion method.

Two cephalosporins, including ceftazidime (30 μg) and cefotaxime (30 μg) combined with clavulanic acid were placed on MHA agar plates. The isolates were positive for ESBL production when the difference of inhibition zone between single ceftazidime (30 μg) or cefotaxime (30 μg) and ceftazidime (30 μg) or cefotaxime (30 μg) combined with clavulanic acid ≥ 5 mm.

### Detection of gene encoding virulence factors and antibiotic resistance

All *E. coli* isolates were determined as pathogenic *E. coli* including Enterotoxigenic *E. coli* (ETEC), Enteropathogenic *E. coli* (EPEC), and Shiga toxin producing *E. coli* (STEC), by specific virulence genes and tested for AMR genes by polymerase chain reaction (PCR). The DNA template was prepared by culturing the bacterial isolate in 5 ml Luria-Bertani (LB) broth (ThermoFisher Scientific, MA, USA) at 37°C overnight while shaking at 120 rpm. The cultured bacteria were harvested and centrifuged at 3,000 rpm for 5 min. The pellet was resuspended with 200 μl sterile distilled water. The suspension was boiled for 10 min and then centrifuged at 5,000 rpm for 8 min. The supernatant containing DNA was collected and used as a template in PCR. PCR reactions containing DreamTaq PCR Master Mix (ThermoFisher Scientific) with a total volume of 25 μl containing 1 μM of the forward and reverse primer each were performed using T100™ Thermal Cycler (Biorad, CA, USA). Primers, annealing temperatures, and positive control isolates used for the detection of virulence genes and antibiotic resistance genes are summarized (Table 1). The PCR amplicon was mixed with StainIN™ GREEN Nucleic Acid Stain (highQu, Kraichtal, Germany), and then separated by 1.5% agarose gel electrophoresis in TAE buffer and visualized using Gel Doc™ EZ Systems (Biorad, CA, USA). The PCR product from positive isolates with no positive control was subjected to nucleotide sequencing and the sequences were analyzed using Nucleotide BLAST for gene confirmation.

**Table 1:** Target genes, positive control isolates, primers, and annealing temperature used in PCR.

Target genes	Positive control	Primer sequences (5’-3’)	Product size (bp)	Annealing temp (°C)	Reference
Virulence genes					
*eaeA*	EPEC	CTGAACGGCGATTACGCGAA	917	60	[24]
		CCAGACGATACGATCCAG			
*lt*	ETEC	TCTCTATGCATACGGAG	322	55	[25]
		CCATACTGATTGCCGCAATT			
*st*	ETEC	TGCTAAACCAGTAGAGTCTTCAAAA	138	55	[26]
		GCAGGCTTACAACACAATTCACAGCAG			
*stx1*	STEC	ACACTGGATGATCTCAGTGG	614	58	[27]
		CTGAATCCCCCTCCATTATG			
*stx2*	STEC	CCATGACAACGGACAGCAGTT	779	58	[27]
		CCTGTCAACTGAGCAGCACTTTG			
Resistance genes					
*bla*_*TEM*_		ATCAGTTGGGTGCACGAGTG	608	60	[28]
		ACGCTCACCGGCTCCAGA			
*bla*_CMY-2_		GACAGCCTCTTTCTCCACA	1,000	55	[29]
		TGGACACGAAGGCTACGTA			
*bla*_OXA_		TCAACTTTCAAGATCGCA	591	58	[30]
		GTGTGTTTAGAATGGTGA			
*qnrB*		GGCATTGAAATTCGCCACTG	264	58	[31]
		TTTGCTGCTCGCCAGTCGAA			
*qnrS*		GCAAGTTCATTGAACAGGGT	428	58	[32]
		TCTAAACCGTCGAGTTCGGCG			
*aadA*		CCCCTGGAGAGAGCGAGATT	152	61	[33]
		CGTGGCTGGCTCGAAGATAC			
*strA*		TGGCAGGAGGAACAGGAGG	405	60	[34]
		AGGTCGATCAGACCCGTGC			
*strB*		GCGGACACCTTTTCCAGCCT	621	58	[34]
		TCCGCCATCTGTGCAATGCG			
*tetA*		GCTGTCGGATCGTTTCGG	658	60	[28]
		CATTCCGAGCATGAGTGCC			
*tetB*		CTGTCGCGGCATCGGTCAT	615	60	[28]
		CAGGTAAAGCGATCCCACC			
*sul1*		CGGACGCGAGGCCTGTATC	591	60	[34]
		GGGTGCGGACGTAGTCAGC			
*sul2*		GCGCAGGCGCGTAAGCTGAT	514	60	[34]
		CGAAGCGCAGCCGCAATTC			
*sul3*		GGGAGCCGCTTCCAGTAAT	500	60	[28]
		TCCGTGACACTGCAATCATTA			
*catA*		CCAGACCGTTCAGCTGGATA	454	58	[28]
		CATCAGCACCTTGTCGCCT			
*cmlA*		TGGACCGCTATCGGACCG	641	58	[34]
		CGCAAGACACTTGGGCTGC			

### Statistical analyses

Prevalence of AMR, MDR, virulence genes, and ESBL producing *E. coli* isolates were determined. Logistic regression analysis was used to determine the association among phenotype and genotype of AMR, virulence genes, and ESBL production. The dependent variable was ampicillin that is commonly used, while the independent variables included resistance phenotype and genotype, virulence genes, MDR, and ESBL production. A *p*-value and confidence intervals were adjusted for potential correlated data within type of market (fresh market and supermarket). Forward selection and backward elimination were used to choose candidates for multivariable analysis. Final regression models based on likelihood ratio test and *p* < 0.05 and two-sided hypothesis testing with 5% of significant level were used. Stata 14.0 (StataCorp, TX, USA) was used to perform all the statistical analysis.

## Results

### Prevalence of fecal coliforms and *E. coli* in Nile tilapia

More than half of the Nile tilapia samples were positive to fecal coliforms and *E. coli* with overall prevalence of 61.6% (510/828) and 53.0% (439/828), respectively, where higher prevalence of both bacteria observed in samples from fresh markets than supermarkets. In fresh market, both fecal coliforms and *E. coli* were commonly found in the intestine (13.8% and 12.9%), followed by meat (12.7% and 10.1%), and liver and kidney samples (12.3% and 9.2%). However, fecal coliforms and *E. coli* isolated from the samples from supermarkets were mostly found in the samples from intestine (11.0% and 10.9%), the second most findings of both bacteria were in liver and kidney (6.6% and 6.0%), and least finding in fish meat (5.2% and 3.9%) (Table 2).

**Table 2:** Prevalence of fecal coliforms and *E. coli* from Nile tilapia.

Source	Type of samples	No. of samples	No. of isolates (%)				
			Fecal coliform	*E. coli*	Resistant *E. coli*^a^	MDR *E. coli*^b^	ESBL- producing *E. coli*^b^
Fresh market	Meat	151	105 (12.7)	84 (10.1)	31 (7.1)	16 (10.1)	0 (0.0)
	Liver and kidney	151	102 (12.3)	76 (9.2)	27 (6.2)	18 (11.4)	4 (2.5)
	Intestine	151	114 (13.8)	107 (12.9)	41 (9.3)	20 (12.7)	4 (2.5)
	Subtotal	453	321 (38.8)	267 (32.3)	99 (22.6)	54 (34.2)	8 (5.1)
Supermarket	Meat	125	43 (5.2)	32 (3.9)	6 (1.4)	3 (1.9)	0 (0.0)
	Liver and kidney	125	55 (6.6)	50 (6.0)	20 (4.6)	12 (7.6)	1 (0.6)
	Intestine	125	91 (11.0)	90 (10.9)	33 (7.5)	17 (10.8)	0 (0.0)
	Subtotal	375	189 (22.8)	172 (20.8)	59 (13.4)	32 (20.3)	1 (0.6)
	Grand total	828	510 (61.6)	439 (53.0)	158 (36.0)	86 (54.4)	9 (5.7)

^a^ calculated from grand total *E. coli* isolates

^b^ calculated from grand total resistant *E. coli* isolates

### Prevalence of phenotypic resistance and ESBL producing *E. coli*

Resistance to antimicrobials was found in 158 *E. coli* isolates. Comparing retails, Nile tilapia collected from fresh markets contained a higher prevalence of AMR *E. coli* (99/158, 62.7%) than those from supermarkets (59/158, 37.3%). The highest proportion of resistance *E. coli* isolates was found in intestine (74/158, 46.8%), followed by liver and kidney (47/158, 29.8%), and meat (37/158, 23.4%) samples, respectively. Forty-eight resistance patterns were found with resistance to only tetracycline as the most common resistant pattern (24/158, 15.2%). Resistance to ampicillin, sulfamethoxazole, tetracycline, and trimethoprim was the second most dominant pattern, but found only in *E. coli* isolates from liver and kidney, and intestinal samples. Interestingly, *E. coli* isolated from meat samples had all of the antibiograms observed in this study (Table 3).

**Table 3:** Distribution of resistant phenotypes of *E. coli* isolates (*n* = 158).

Resistance phenotypes	No. of resistant *E. coli* isolates (%)			
	Meat samples (*n* = 37)	Liver and kidney samples (*n* = 47)	Intestine samples (*n* = 74)	Total (*n* = 158)
AMP	25 (67.6)	33 (70.2)	57 (77.0)	115 (72.8)
CHL	8 (21.6)	3 (6.4)	14 (18.9)	25 (15.8)
CIP	14 (37.8)	8 (17.0)	9 (12.2)	31 (19.6)
GEN	4 (10.8)	3 (6.4)	3 (4.1)	10 (6.3)
STR	6 (16.2)	18 (38.3)	21 (28.4)	45 (28.5)
SUL	16 (43.2)	24 (51.1)	32 (43.2)	1 (0.6)
TET	28 (75.7)	37 (78.7)	59 (79.7)	124 (78.5)
TRI	16 (43.2)	22 (46.8)	31 (41.9)	69 (43.7)

The full name of tested antibiotics was abbreviated: AMP, ampicillin; CHL, chloramphenicol; CIP, ciprofloxacin; GEN, gentamicin; STR, streptomycin; SUL, sulfamethoxazole; TET, tetracycline; TRI, trimethoprim.

More than half of the resistant *E. coli* isolates were MDR (86/158, 54.4%), while the fresh markets (54/86, 62.8%) had higher MDR proportion than supermarkets (32/86, 37.2%). The MDR *E. coli* were mainly isolated from intestinal samples (37/86, 43.0%), followed by liver and kidney samples (30/86, 34.9%), and meat samples (19/86, 22.1%) respectively. ESBL producing *E. coli* prevalence was at 9/158, 5.7%, and was not detected in any meat samples (Table 2).

### Genotypes of pathogenic and AMR *E. coli*

**Fig 1:**
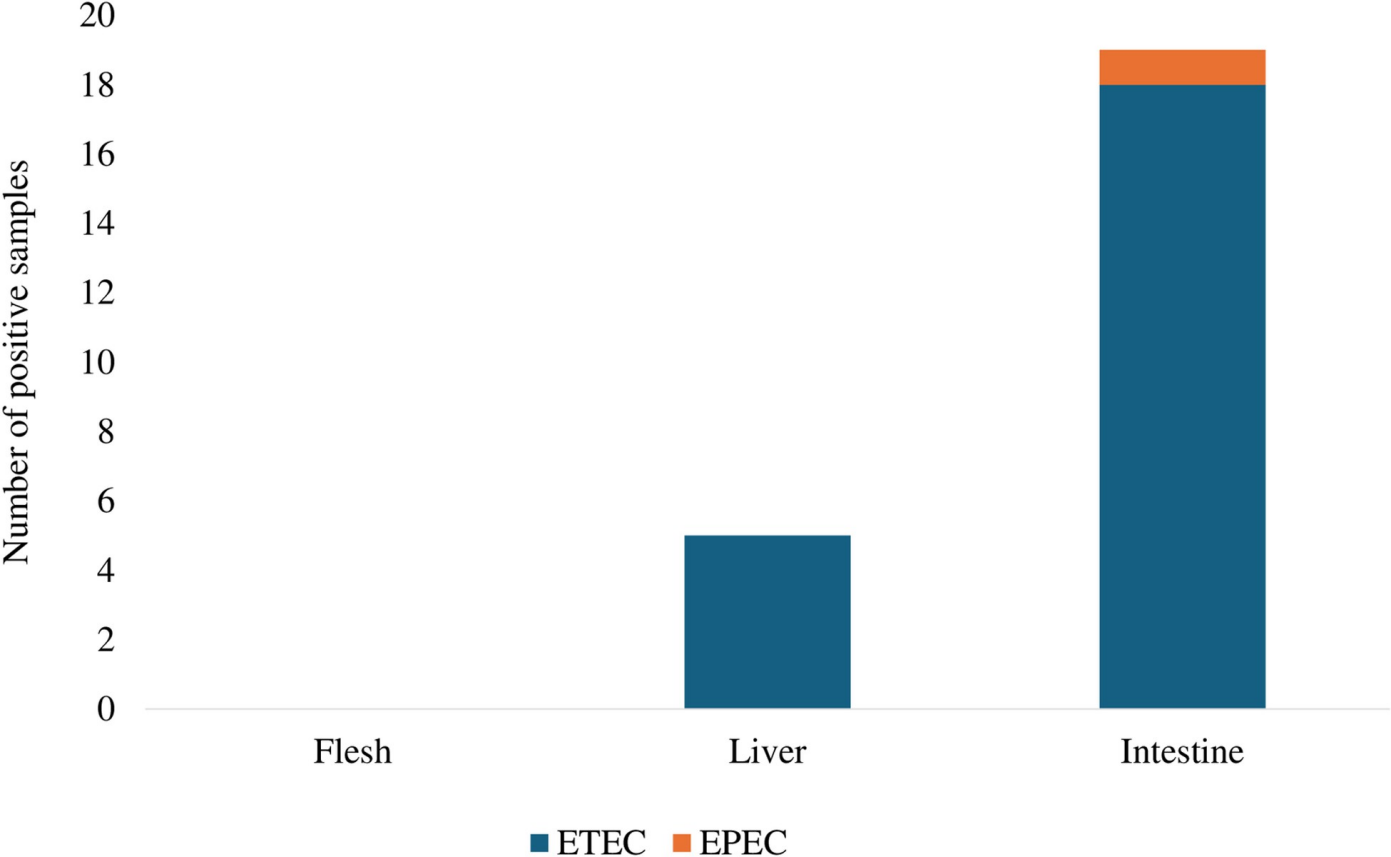
Distribution of pathogenic *E. coli* in Nile tilapia samples.

Genotypes of pathogenic *E. coli* using specific virulence genes revealed that ETEC was the most abundant pathogenic *E. coli* strains found in liver and kidney, and intestinal samples. EPEC was found in one intestinal sample, and none of pathogenic strains were found in any isolates from meat (Fig 1).

**Fig 2:**
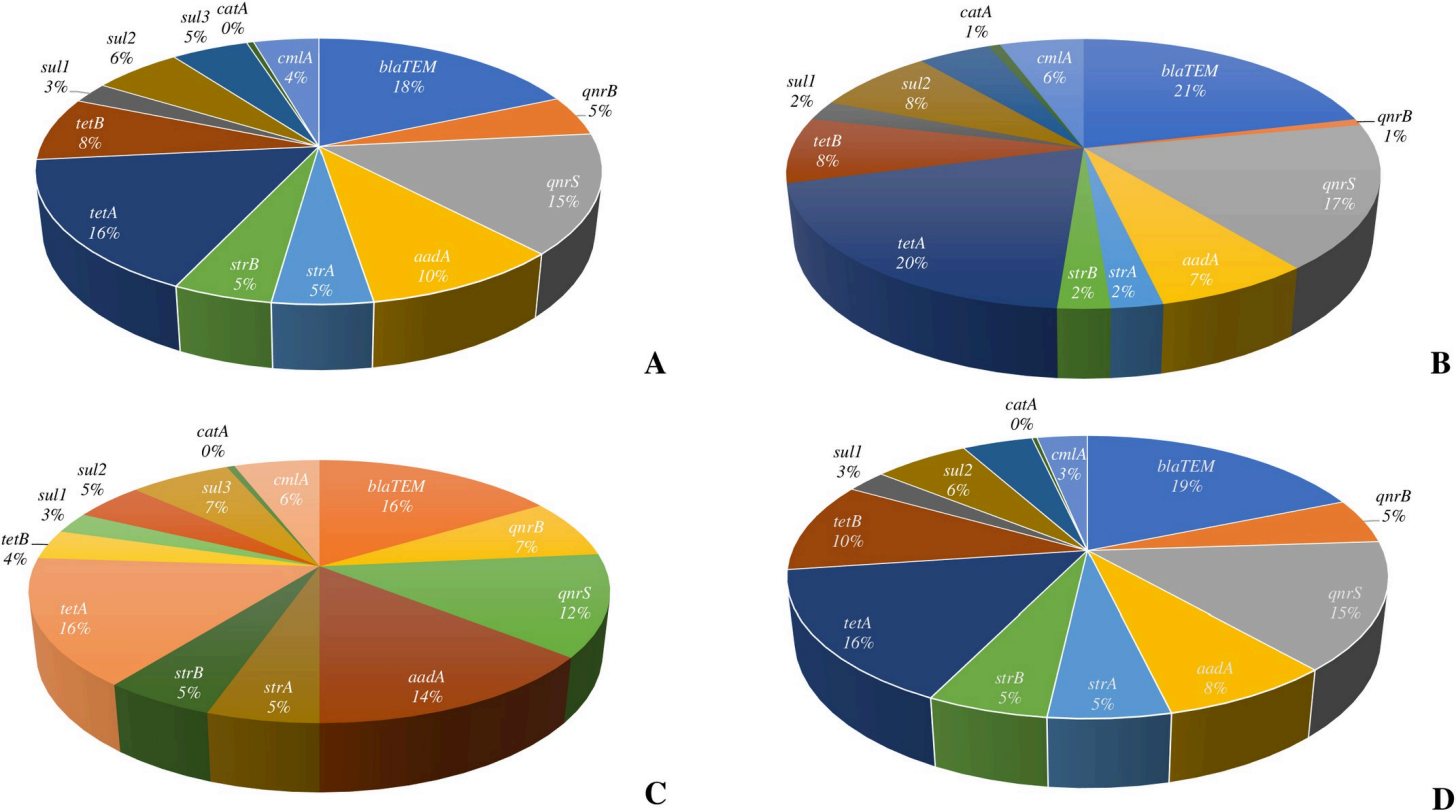
Genotyping results from different sample types. (A) Overall genotyping from all sample types, (B) Genotyping results from meat samples, (C) Genotyping results from liver and kidney samples, (D) Genotyping results from intestine samples.

**Fig 3:**
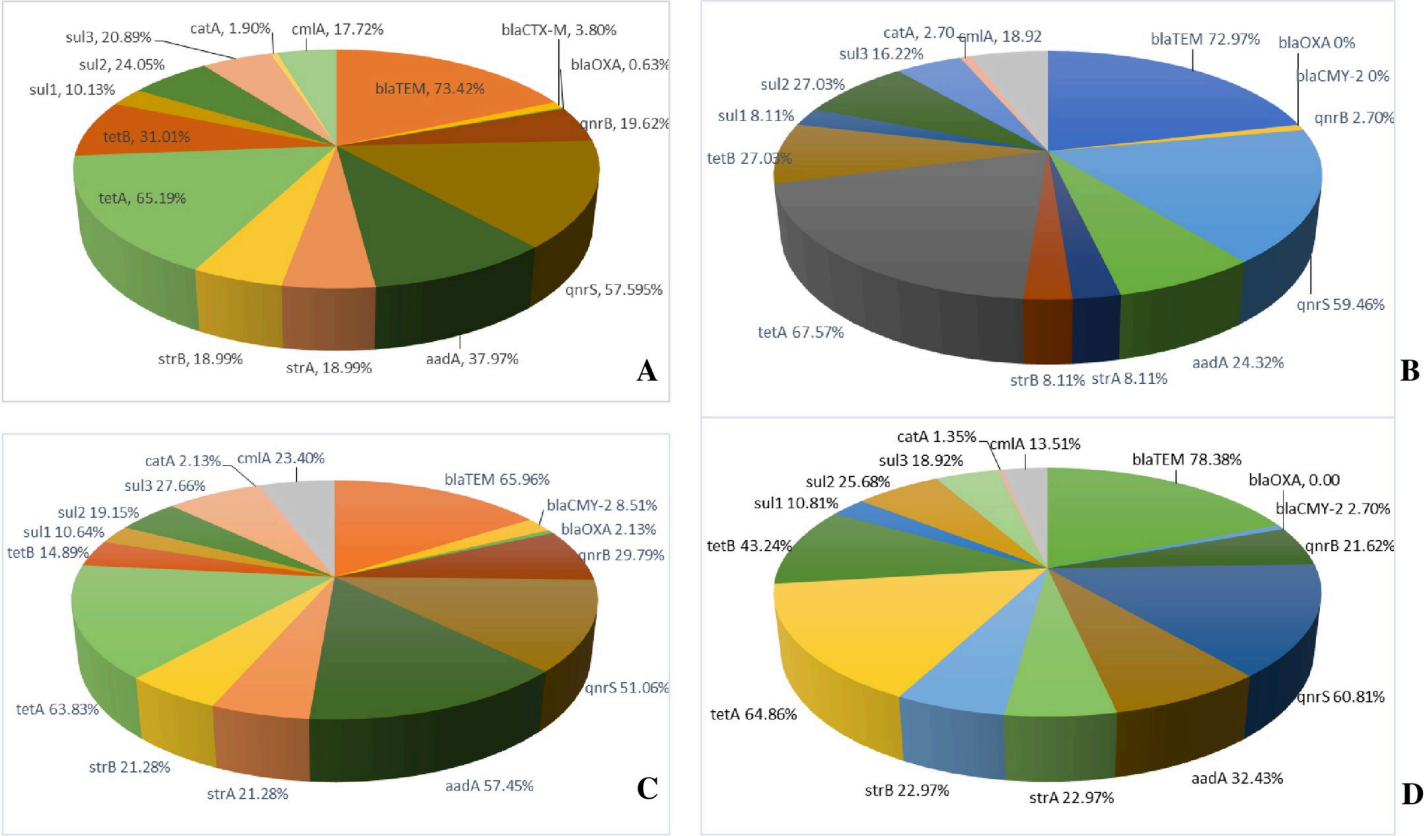
Distribution of resistant *E. coli* isolates with correlated AMR gene.

The most dominant AMR gene found throughout the resistant *E. coli* isolates was *tetA* (103/158, 65.2%), followed by *qnrS* (93/158, 58.9%) (Fig 2). These genes were also the most abundant in all types of samples. Other dominant resistant genes were *aadA* found in *E. coli* isolates from liver and kidney (27/47, 57.4%), and intestine samples (24/74, 36.5%), while *tetB* was the third most dominant resistant gene in meat isolates (10/37, 27.0%) and intestinal samples (32/74, 43.2%). Interestingly, the genotypes of all resistant isolates did not fully correspond with the phenotypes. Only 62.0% (98/158) of the resistant isolates, their existing genotypes corresponded to the resistant phenotype (Fig 3). Less than half of the meat sample isolates (16/37, 43.2%), the harbored AMR genes corresponded to the phenotypic resistance.

The most dominant ESBL related gene throughout all types of samples was *bla*_TEM_ (116/158, 73.4%). The proportion of *bla*_CMY-2_ was 6/158 (3.8%), and *bla*_OXA_ was 1/158 (0.63%), respectively (Fig 2). The proportion of *bla*_TEM_ in ESBL positive isolates from all types of samples is highest in the intestinal sample (58/74, 78.38%), followed by meat samples (27/37, 72.97%), and liver and kidney (31/47, 65.96%), respectively (Fig 2). Meat samples showed no presence of *bla*_CMY-2_, but the gene was found in liver and kidney (4/47, 8.51%), and intestinal samples (2/74, 2.70%) (Fig 2). Interestingly, only one ESBL positive isolated from liver and kidney sample harbored *bla*_OXA_.

### Multivariable logistic regression analysis

Phenotypic resistance to tetracycline and trimethoprim and the presence of *bla*_TEM_ and *tetA* were significantly associated with the presence of ampicillin-resistant isolates (Table 4). A positive association was observed among *E. coli* isolates resistant to ampicillin and resistant to tetracycline and trimethoprim and the presence of *bla*_TEM_, while a negative association was examined in those isolates harboring *tetA* gene. This suggested that there is a mechanism linked to the resistance of ampicillin, tetracycline, and trimethoprim. However, inconsistency between phenotypic and genotypic resistance to tetracycline was determined among ampicillin-resistant isolates.

**Table 4:** Logistic regression model for factors associated with ampicillin-resistant *E. coli* in Nile tilapia.

Factor	Coefficient	Robust Std. Err.^a^	95% C.I.^b^	*p*-value
tetracycline	3.232	1.173	0.931 to 5.533	0.006
trimethoprim	3.408	1.500	0.467 to 6.350	0.023
*bla*_TEM_	5.584	0.090	5.409 to 5.760	<0.0001
*tetA*	-1.986	0.567	-3.097 to -0.875	<0.0001
Constant	-4.601	0.334	-5.255 to -3.947	<0.0001

Akaike Information Criteria (AIC): 70.03

^a^ Robust Standard Error.

^b^ Confidence Interval.

## Discussion

Nile tilapia has been widely consumed worldwide, therefore intensive aquaculture farming system has been implemented to serve high demand both nationally and internationally. Even though Thailand is one of the major exporters of aquatic products [17], the epidemiological study of AMR and virulence genes in aquaculture is still limited. To strengthen AMR monitoring and surveillance in aquaculture, characterization of the phenotypic and genotypic resistance, ESBL production, and virulence genes of *E. coli* isolated from Nile tilapia was performed.

*E. coli* has been commonly used as priority organism to monitor AMR in humans and animals. The contamination of resistant *E. coli* in Nile tilapia can occur from fish production site through to consumption. This study emphasized the high prevalence of fecal coliforms and *E. coli* contamination in Nile tilapia sold in fresh markets and supermarkets. Various sources of potential contamination of AMR *E. coli* should be carefully monitored throughout the production pipeline, as it could occur at any stage. The contaminant from the farm may arise from fecal material in water or soil used in the raising pond. During transportation and in fresh market, the contamination may originate from dust, ice or storage water. In the selling area, contaminants could even be found on knives or the display table. The result also indicated a high prevalence of AMR *E. coli* in intestine of the fish, therefore the contamination of AMR *E. coli* in fish flesh might come from the intestine during the preparation process. Further investigation is needed to assess the hygienic practices of the handler and the bacterial contamination on tools and surfaces used in the selling area. This is crucial for determining the sources of contamination and associated risk factors, leading to a better understanding, and improved planning for prevention and control measures. The overall prevalence of fecal coliforms and *E. coli* were higher in fresh markets (fecal coliforms 38.8%; *E. coli* 22.8%) compared with supermarkets (fecal coliforms 32.3%; *E. coli* 20.8%). These findings agreed with previous studies that high prevalence of *E. coli* in Nile tilapia was observed [35, 36]. In addition, the result was similar to previous study in Thailand, which reported high prevalence of *E. coli* and *K. pneumoniae* collected from fish markets compared to fish farms [37]. Higher prevalence of *E. coli* in fresh markets can reflect inappropriate storage, cross-contamination, or poor hygienic condition. At farm level, contaminated water or cross-contamination during harvest and transportation can contribute to inconsistent levels of *E. coli* [38, 39].

The highest prevalence of *E. coli* was observed in intestinal samples in Nile tilapia from both fresh markets and supermarkets in this study. The findings agreed with the study in Egypt [40]. This study reported EPEC was found in intestine sample only (Fig 1), which was in accordance with the studies reported high prevalence of EPEC in fish sold in fresh markets in Brazil [41, 42]. Very few studies have observed the presence of ETEC in Nile tilapia, but this study reported a high prevalence of ETEC in intestine, and liver and kidney samples (Fig 1). This is concerning since the consumption of tilapia contaminated with ETEC can lead to diarrhea, even though the symptoms are not life threatening. Interestingly, the high prevalence of *E. coli* found in fish meat was determined in this study, however, none of them were pathogenic *E. coli*. Regardless that fish meat has been noted as a sterile sample, the presence of *E. coli* in fish meat in this study may be because microorganisms can penetrate from degradation of digestive system to muscle fiber when fish are restrained and caught during harvest (40). From food safety perspective, the national and international limits of *E. coli* in fish for human consumption should not exceed 10 MPN/g [43, 44]. However, this study failed to quantify the level of *E. coli* in meat samples. Therefore, the enumeration of fecal coliforms and *E. coli* should be further investigated for public health purposes.

The contamination of AMR bacteria in Nile tilapia in retails is of public health significance. Nile tilapia is typically cultured in an earth pond, relying on water from local canals for pond filling, draining, and maintaining water level of the pond through water inlet and outlet. However, the low concentrations of antimicrobials in the pond and canal water indicated that the effectiveness of Thailand policies of antimicrobial use in aquaculture. Rather than focusing solely on the level of antimicrobial usage, attention has been directed towards the correlation between fecal bacteria and genes resistant to antibiotics, revealing important insights [45]. Therefore, the contamination of the AMR *E. coli* in Nile tilapia was likely to originate from human *E. coli* and pollution from inadequate water treatment which contaminated in the canals water [46]. Occurrence of AMR in tilapia had been reported worldwide [35]. Antimicrobial use in humans, and animals, including aquaculture can drive the bacteria resistance. Improper use of antimicrobials as growth promotors and prophylaxis in aquaculture has been documented, and evidence suggested that it might lead to AMR accumulation in the fish and surrounding environment [47, 48]. Despite limited evidence of antimicrobial use in Nile tilapia [49], this study reported the prevalence of resistant *E. coli* isolates at 36.0% and high prevalence of MDR *E. coli* at 54.4%. Many studies have shown that most of the bacteria, especially *E. coli* and *Salmonella* spp. isolated from tilapia are MDR [48, 50, 51]. Generally, antimicrobial use in aquaculture is varied in each region depending on local regulations. This study reported high resistance to tetracycline, beta-lactam, and sulfamethoxazole, which correlated with antibiotic groups highly use in aquaculture in Thailand [52]. The resistance pattern was similar to the study of AMR *E. coli* isolated from tilapia collected directly from aquaculture farms in Malaysia [51]. This might indicate the fish cultivation sites as one of the major sources of resistant organisms. However, it is worth noting that certain resistance genes can be acquired naturally by bacteria regardless of the use of antibiotics [53]. Therefore, the result calls for consistently monitoring of AMR *E. coli* and other pathogenic bacteria in aquaculture. The low prevalence of ESBLs-producing *E. coli* was less than 6.0%, which was in agreement with the low prevalence of ESBLs-producing *E. coli* reported in tilapia in Vietnam when compared with higher prevalence of ESBL *E. coli* in other fish species [50]. Most of the ESBLs-producing *E. coli* isolates were MDR, but none of the isolate were pathogenic *E. coli*. ESBLs-producing *E. coli* isolates that harbored resistance and MDR genes are of public health threat, since these isolates can facilitate resistance to other bacterial species impacting dissemination and evaluation of AMR in the environment [54]. Even though the prevalence is low, surveillance of the ESBLs-producing bacteria should be conducted regularly and consistently to monitor the culture standard and prevent the spread of the bacteria.

Based on logistic regression analysis, the ampicillin-resistant isolates were more likely to resist tetracycline 25.3-fold (OR = 25.3, e^3.232^, *p* = 0.006) and trimethoprim 30.2-fold (OR = 30.2, e^3.408^, *p* = 0.023) than those susceptible isolates. In addition, the *E. coli* isolates showing ampicillin resistance were more likely to contain *bla*_TEM_ gene (OR = 266.3, e^5.584^, *p* < 0.0001). This finding was supported by previous study indicating an interaction between β-lactamase genes (*bla*_TEM-1_) and tetracycline efflux pump, which generally encoded on transmissible elements, including plasmid, integrons, and transposons [55]. It is evidence that co-selection of ampicillin and trimethoprim can occur through mobile genetic elements [56]. However, this study observed the negative association between ampicillin resistance and the presence of *tetA* genes (OR = 0.137, e^-1.986^, *p* < 0.0001), meaning that the ampicillin-resistant isolates were less likely to confer *tetA* 7.30 times (1/0.137 = 7.30) than those susceptible ones. This result addressed that even though the AMR is usually involved in genetic changes, the result showed that genotype of AMR *E. coli* isolates did not correspond well with their resistant phenotypes. Evidence from past studies reported similar results in *E. coli* and *Salmonella* spp. [33, 57]. This might be the result of limitation of the detection method by PCR. However, the resistance of the bacteria without corresponding genotype is existed and labelled as solely phenotypic resistance, which is usually found during infection process due to the stationary growth phase or persistence [58]. However, the phenomenon is not considered in the antimicrobial susceptibility test. This suggested that there might be other genes or regulatory factors that influence resistant phenotype of the bacteria. To overcome the limitation and explore other genes in the bacterial genome, whole genome sequencing using next-generation sequencing might be able to give all information on the resistance genes in the bacteria. Resistant pathogenic *E. coli* in Nile tilapia can contribute to a difficult to treated gastrointestinal infection in consumers. Other resistant *E. coli*, on the other hand, can become a reservoir of the resistant genes and transfer the genes to other pathogenic bacteria via horizontal gene transfer [59, 60]. The presence of AMR *E. coli* in Nile tilapia in the retail is concerning since they can serve as reservoir of resistant genes and disseminate them to other bacteria. Hence, AMR surveillance and monitoring system in the environment, observation of AMR in aquatic animals sold in markets is needed.

## Conclusion

Taken together, this study addressed the circumstance of circulated resistant *E. coli* in Nile tilapia sold in fresh markets and supermarkets in Thailand. This study identified that fish intestine was a dominant source of fecal coliforms and *E. coli* contamination and could be used as a target sample for AMR study in aquaculture. Pathogenic and resistant *E. coli* contaminated in Nile tilapia can pose a serious public health threat. This result can be used to support further investigation to identify the potential source of resistant bacterial contamination. Study of AMR in aquaculture under One Health is also needed to generate baseline epidemiological data for effectively prevent and control AMR. Furthermore, implementation of better sanitation and control spread of AMR bacteria in the environment are recommended. Continuing monitoring and surveillance of AMR will assist to strengthen AMR national strategic plan in aquaculture.
